# Influence of Washing and Sterilization on Properties of Polyurethane Coated Fabrics Used in Surgery and for Wrapping Sterile Items

**DOI:** 10.3390/polym12030642

**Published:** 2020-03-12

**Authors:** Beti Rogina-Car, Stana Kovačević, Suzana Đorđević, Dragan Đorđević

**Affiliations:** 1 Department of Clothing Technology, University of Zagreb Faculty of Textile Technology, Prilaz baruna Filipovića 28a, 10000 Zagreb, Croatia; 2 Department of Textile Design and Management, University of Zagreb Faculty of Textile Technology, Prilaz baruna Filipovića 28a, 10000 Zagreb, Croatia; stana.kovacevic@ttf.unizg.hr; 3 Textile Department, Higher Technological and Artistic Professional School Leskovac, Vilema Pusmana 17, 16000 Leskovac, Serbia; szn971@yahoo.com; 4 Textile Department, Faculty of Technology, University of Nis, Bulevar Oslobodjenja 124, 16000 Leskovac, Serbia; drdrag64@yahoo.com

**Keywords:** medical textile, polyurethane coated fabrics, microbial barrier, microorganisms, FTIR

## Abstract

The objective of this work was to determine the influence of washing and sterilization under real hospital conditions on properties of microbial barrier offered by polyurethane coated fabrics used in surgery and for wrapping sterile items. Emphasis was put on the change of surface polyurethane coating by using FTIR analysis. The permeability and durability of the microbial barrier were determined after 0, 10, and 20 washing and sterilization procedures according to previously developed methods. Bacterial endospores of the apathogenic species of the genus Bacillus *Geobacillus stearothermophilus* and *Bacillus atrophaeus* were used. Mechanical damage to medical textiles in the washing and sterilization process was determined according to standard HRN EN ISO 13914-1:2008 and associated with changes in physical and mechanical properties. Chemical changes of PU coatings were determined using FTIR analysis. The results showed an exceptionally efficient microbial barrier and its durability in all samples after 0, 10 and 20 washing and sterilization procedures and for a period of one, two and three months.

## 1. Introduction

Coated fabrics are defined as technically flexible composite materials coated with a polymer coating on one or both sides. The substrate, to which a polymer coating is applied, can be woven fabric, knitted fabric or nonwovens. Coating polymers are applied onto the surface substrate as viscous solutions or dispersions. After applying the polymer coating, the liquid phase is removed by heat and a continuous polymer coating is formed on the fabric surface. The most used polymers for textile coating are natural and synthetic rubber, polyurethane (PU), polyvinyl chloride, polyacryl and polyvinyl acetate. PU is a multi-purpose coating polymer used for coating protective clothing items [1,2,3,4,5]. The properties of coated fabrics are a combination of the properties of the base fabric and the polymer coating applied to the fabric. The result of this combination of properties provides many properties that individual components cannot offer, so careful consideration should be given to selecting the base fabric and polymer coating. Thus, it is necessary to carefully consider the selection of the base fabric and polymer coating. The substrate or the base fabric gives the mechanical strength of the composite material and supports the coating applied onto it. High quality base woven and knitted fabrics are necessary to obtain high quality coated fabrics [1]. The PU polymer coating provides fluid tightness and the ability to pass water vapor from the body to the environment. The knitted fabric structure allows for elasticity and flexibility, but it has a high porosity due to its specific structure [6,7,8]. Polyurethane is a material that is acceptable for medical use because of its excellent elasticity and biocompatibility. The microporous structure allows breathing and passing of the sterilizing media. The literature reveals the broad use of polyurethane in medicine [9,10,11,12,13,14,15,16]. Medical textiles belong to the group of technical textiles which can be used in medicine. This group of textiles is one of the most strictly controlled, but also most complex in meeting the set conditions. Woven and knitted fabrics are the most present of all textile materials in all segments of medicine. They meet basic requirements in the protection of persons and objects due to their comfort, as well as due to their strength and stability. Higher efficiency of textile materials is achieved by selecting the structure and surface treatment. Multiple washing and sterilization damages textile materials, which limits their use [17]. In order for woven and knitted fabrics to meet medical standards, they often have to satisfy some of the basic requirements, according to their purpose, such as: being impermeable to microorganisms; biocompatible, while at the same time drug compatible; able to have chemical and thermal sterilization; non-toxic, non-allergic, non-carcinogenic; they must have good physical and mechanical properties; stability of shapes and dimensions; sometimes permeable to x-rays; must have good absorption properties of liquids and secretion; must meet standards and regulations according to their purpose; possibility for their rational and economical production, etc. [18]. Washing or dry cleaning and sterilization make them reusable. Woven and knitted fabrics that have been surface-treated, sterilized and made microbe impermeable are often reused for cost reductions. Their lifespan depends on the effectiveness of the protection against microorganisms and on mechanical properties [19]. The function of medical textiles is to protect against bacteria and viruses originating from staff and patients. Surgical drapes, compresses and sterile sheets are used to cover the surgical incision environment, thus separating the anesthesiological space from the surgical field, the purpose of which is to prevent the transfer of bacteria from the skin to the wound. Surgical drapes must provide adequate protection against passage of microorganisms. Woven and knitted fabrics are also used as covers for sterilized instruments before use in operating rooms. Therefore, it is important to examine mechanical damages and their impact on barrier properties. Covers must be free of toxic ingredients and retain integrity, durability and withstand physical conditions of standard stress during use [20,21,22,23].

Microbial barrier is a basic packaging or a container that protects the medical material after sterilization from recontamination. The microbial barrier system should provide protection against the penetration of microorganisms and maintain the sterility of products until they are used. The properties of the microbial barrier in medicine must be present in wraps for wrapping sterilization packages, surgical gowns, hospital sheets, compresses and other surgical scrubs. Woven and knitted fabrics are used in sterilization for internal wrapping instruments or for external dust protection. They should be subjected to a washing process before use. The pores of woven and knitted fabrics are mostly larger than microorganisms, so manufacturers do not recommend them as an adequate barrier, if they are single-layered and without surface treatment. Despite that, woven and knitted fabrics are the most common textile materials used in medicine. The first choice of partial substitution are paper sheets used for primary packaging. They have smaller pores than woven and knitted fabrics and can be used for primary packaging, but only once [24,25,26]. The requirements to be met by packaging materials as well as the conditions for handling these materials are determined by standard EN ISO 11607 “Packaging of medical instruments and sterilization equipment” [27,28]. The term “packaging system” means a combination of a microbial barrier system (SMB) and a protective packaging. The most important function of the packaging is the protection of sterile material from contamination after sterilization. The packaging system must be suitable and durable. It must not adversely affect the efficiency of the sterilization process. Particular attention should be paid to the mass, outer shape, sharp edges or parts of protruding instruments and accessories. The contents of a specific package are applied only to one patient and in the same procedure, and everything else is considered as non-sterile. By selecting the appropriate packaging system, medical instruments and accessories may be packaged, sterilized in packaging, shipped and stored in a sterile state before use, and removed from the packaging without contamination [25,26].

Polyurethane (PU) is one of the multiple polymeric materials widely used in various fields and also in medicine for medical synthetic materials and compresses. Polyurethane coatings typically contain TiO_2_ (rutile) as a UV blocking agent and are widely used in various industries due to their durability and generally good balance of mechanical properties [29,30,31]. Antimicrobial PU coatings have been developed for various applications to reduce the proliferation of bacteria, fungi and viruses on surfaces. The latest scientific research involving antibacterial coatings for textiles attracts attention in the health industry due to the increased risk of health-care associated infections [32]. The priority of each health facility is to reduce the number of pathogenic microorganisms in the patient’s environment. Antimicrobial agents are integrated into textile materials in several ways such as: spin coating, dip coating, layer-by-layer coating, or spray coating [33]. Various methods are available to improve the durability of the finishing process. For example, attaching through chemical bonding of the fiber and the use of graft polymers, homopolymers, and/or copolymerization onto the fiber, products of various available functional groups of the polymer, insolubilize the active substances in/on the fiber, cross-linking agents, enclose the bactericidal agents with the fiber/ polymer matrix, coating the fiber surface [34,35].

The objective of this work was to determine the effect of washing and sterilization on the dry permeability of microorganisms of medical polyurethane coated textile materials. The aforementioned material is used in operating rooms and for packaging surgical sterilization materials in the medical sterilization unit. Special emphasis was given to the polyurethane coating and its changes occurring after 0, 10 and 20 procedures of washing and sterilization in real hospital conditions. The durability or retention period of the microbial barrier of sterilized diagonally packed packages (one coating; EN ISO 11607-1 2009) was determined after storage for a period of 1, 2 and 3 months in real hospital conditions. Changes in mechanical properties after use in real conditions were identified. Changes in the surface polyurethane coating was determined by using FTIR analysis.

## 2. Materials and Methods

### 2.1. Materials

Four samples were used made by the Čateks Texile Company, Čateks, Croatia, Table 1. The surface coating was made using the polyurethane coating technology and treated with an additive preventing the growth and development of bacteria and fungi. Woven or knitted fabrics without antibacterial treatment were used on the back sides of samples.

The fabric was woven in plain weave (WF_MC), the other three samples are single jersey fabric (KF_M) and double jersey fabric (KF_B, KF_S), Table 1. The test samples shall be used in medicine. Raw materials and mass per unit are different, while the polyurethane coating has the same composition and thickness. Knitted fabrics differ from each other by the density of courses and wales, which changes the mass per unit area.

The samples of polyurethane coated fabrics used for medical application were made using the polyurethane coating technology for coating woven and knitted fabrics. A PU coating was applied to the substrate (woven or knitted fabric) by the indirect transfer process, first to the paper, and then by laminating the substrate, followed by drying, cooling and finally separating the paper from the finished material [36,37].

**Figure 1:**
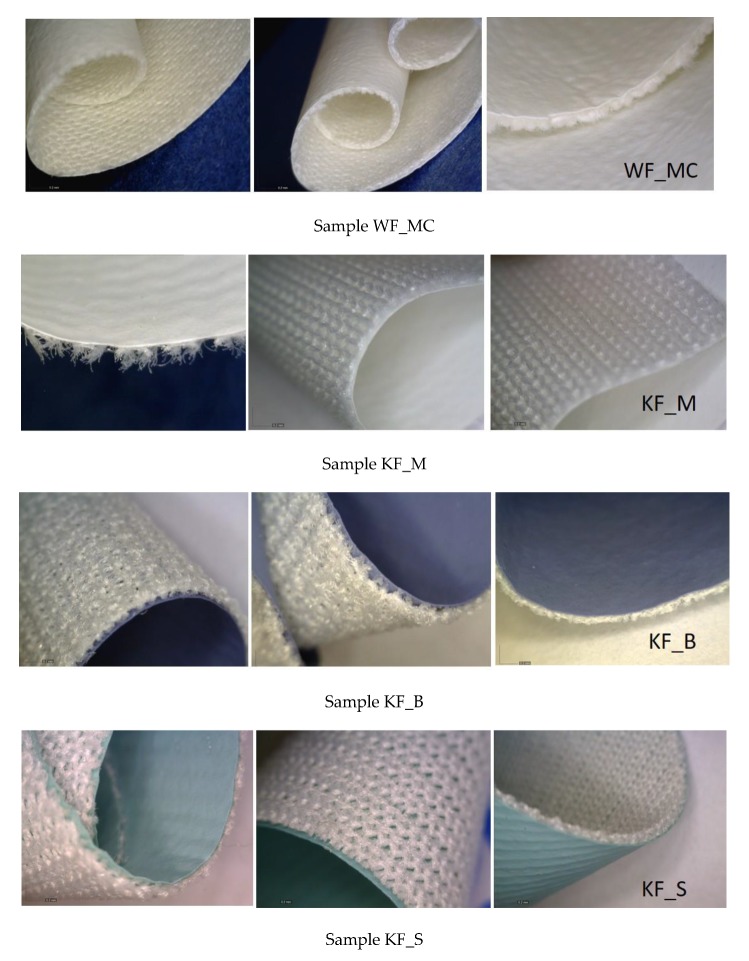
Polyurethane (PU) laminate surface.

Figure 1 shows the surfaces of PU laminates, i.e., their appearance on the face side (PU coating) and on the back side (substrate: woven fabric of the sample WF_MC, and the knitted fabrics KF_M, KF_B, and KF_S).

### 2.2. Methods

#### 2.2.1. Washing and Sterilization

Washing and sterilization procedures were performed in the specialized laundry of the University Hospital Center Zagreb, Rebro. Medical textiles were washed in a continuous washing machine (JENSEN brand, Gent, Belgium) according to a specially defined procedure, Table 2. The samples were sterilized in the Selectomat PL MM hospital steam sterilizer (Münchener Medizin Mechanik, Planegg, Deutschland) at 134 °C and at a pressure of 2.5 bar for 5 min.

Dimensional stability was determined according to the standard test procedure in HRN EN ISO 6330 2003 and HRN EN 25077 2003. In order to determine the change in dimensions after the washing and sterilization procedures, the length, the width and the initial dimension of 25 × 25 cm (625 cm^2^) were marked on the samples. After the washing and sterilization procedures, sample measurements were performed again and the percentage changes in sample size and sample surface were calculated.

Microscopic analysis of the polyurethane coating was performed using Mesdan Video analyzer 250D (manufacturer MESDAN S.p.A., Brescia, Italy, 2014).

#### 2.2.2. Microbial Barrier Properties

**Figure 2:**
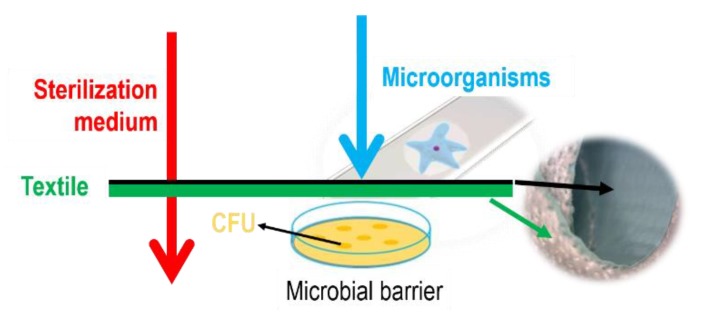
Microbial barrier.

The microbial barrier system should provide protection against the penetration of microorganisms and maintain the sterility of products until they are used, Figure 2. One of the main requirements for medical textiles used in sterilization process is to ensure its permeability for sterilizing media. The procedure of sterilization is performed in a steam sterilizer at 134 °C for 5 min at a pressure of 2.5 bar (3.04 bar absolute pressure). This treatment is the main reason for permeability of PU-coated textile, as it allows the passage of sterilization media, but not the passage of the microorganisms.

**Figure 3:**
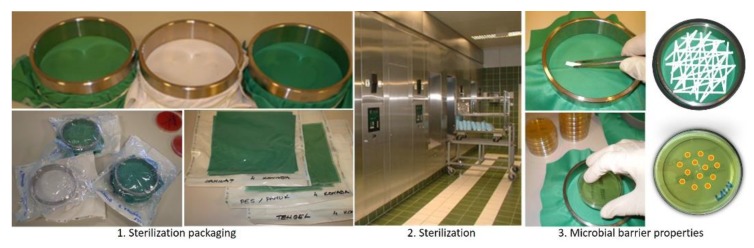
Microbial barrier properties.

The purpose of this research was to test the effectiveness of the microbial barrier system polyurethane coated fabrics unwashed and after 10 and 20 washing processes and 10 and 20 sterilization cycles in real hospital conditions [38,39]. Textile samples were prepared by fixing them on an O-ring device and then placed in transparent packaging. The packaged samples were subsequently exposed to sterilization at 134 °C for 5 min, after which the packaging was opened in a sterile environment to prevent contamination. In aseptic conditions, the spores were rubbed in equal motions on the face side of the tested samples. The procedure was then repeated in the same order with the biological indicator rod reversed. A print was taken using a CT3P agar print plate (bioMe’rieux SA, Marcy I’Etoile, France), first from the back side, and then from the face side, with a new plate. Agar plates were incubated for 72 h at 35 °C, after which colony forming units (CFUs) were counted, Figure 3.

This newly developed method of testing microbial barrier permeability in dry conditions involves directly rubbing the microorganisms onto the sterilized samples [38]. In the tests the most resistant microorganisms of the bacterial endospore of apathogenic species of the genus *Bacilllus Geobacillus Stearothermophilus* and *Bacillus Atrophaeus* were used.

#### 2.2.3. New Method for Testing the Durability of Microbial Package Barrier after Sterilization

**Figure 4:**
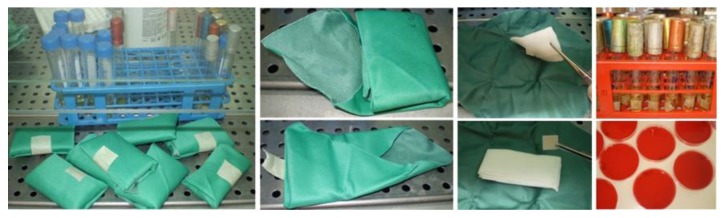
Sterilized packs stored in the warehouse for a period of 1 and 2 months. Presentation of the inside of the pack: gauze and filter paper “Whatman No 1” with a dimension of 1 cm^2^ which is put into a test tube with brain–heart broth after which it is planted on blood agar.

After 10 and 20 washing procedures the tested samples of dimensions 22 × 22 cm were packed diagonally according to the defined scheme. The gauze, under which filter paper “Whatman No 1” of a dimension 1 cm^2^ is placed, is packed into packs (Figure 4).

The packs packaged in this way undergo the sterilization process (134 °C for 5 min) and are stored in a protected warehouse under the following microclimatic conditions: temperature 15–30 °C, relative humidity 30–60%. The tested materials are stored on shelves according to the rule: distance from the floor 25 cm, from the ceiling 45 cm, from the walls 5 cm. The storage time of packs lasts for 1, 2 and 3 months. After a specified storage time, packs are removed from the warehouse, unwrapped in sterile conditions, and sterile tweezers are used to take the absorbent paper, which is then put into a test tube with brain–heart broth, Figure 4. After an incubation of 48 h at a temperature of 35 °C, a change in the clarity of the broth is observed if the turbidity occurred. Sterility is additionally checked by planting on a solid nutrient medium (blood agar). A calibrated inoculation loop is used to pipette 0.5 mL of brain–heart broth, and it is planted on blood agar. It is then incubated for 48 h at 35 °C. After 48 h the number of bacterial colonies is read [38].

#### 2.2.4. Breaking Strength and Breaking Elongation

Tensile strength tester (Tensolab Mesdan S.P.A., Brescia, Italy) was used to evaluate mechanical properties through breaking strength and breaking elongation of the initial, washed and sterilized samples after 0, 10 and 20 cycles according to the standard EN ISO 13934-1 [40]. During strength tests according to the standard, the test material is exposed to a uniform linear tensile force. It increases constantly until the material is broken. The higher the determined value is at the moment of breaking, the more stable the material is.

#### 2.2.5. Fourier-Transform Infrared Spectroscopy-FTIR

The FTIR analysis of samples—potassium bromide technique, spectrophotometer BOMEM Hartmann and Braun MB-Series (ABB Group, Zurich, Switzerland) in the range of wavelengths 4000–400 cm^−1^ was conducted.

## 3. Results and Discussion

The constructional parameters of fabrics were tested according to the following standards: ISO 3801 (fabric mass), ISO 5084:1996 (fabric thickness) [41,42]. The results in Table 2 show a change in mass per unit area and thickness after washing and sterilization processes performed under real hospital conditions. It was found that washing and sterilization processes caused the shrinkage of the tested textile samples, resulting in an increase in the fabric thickness.

The washing and sterilization procedures caused a change in dimensions in almost all samples. Dimensional changes are shown in percentage values with a negative sign, which means that all samples have shrunk. Slight changes were seen on the sample (WF_MC) with the woven fabric in the substrate only after 20 washes and sterilization. Only the sample WF_MC had 100% cotton woven fabric base, that has proved to be more stable and less prone to shrinking during the washing and sterilization process. The sample was exposed to mechanical effect, during the washing and sterilization process, which caused the surface of the polyurethane coating to become rougher, resulting in an increase in thickness while maintaining the same mass.

After only 10 washing and sterilization, the samples with the knitted fabric shrank significantly. After further washing no shrinkage of the samples with the polyester knitted fabric in the substrate was observed (samples KF_M and KF_S). Sample KF_B with the polyamide knitted fabric continued to shrink even after 10 washes. Sample KF_S with the polyester fabric and the greatest thickness had the highest shrinkage.

Finally, the samples with the polyester knitted fabric had the highest shrinkage that stabilized after 10 washes, while the sample with the polyamide knitted fabric shrank less but needed more washing and sterilization cycles to achieve dimensional stability. The results are listed in Table 3.

**Figure 5:**
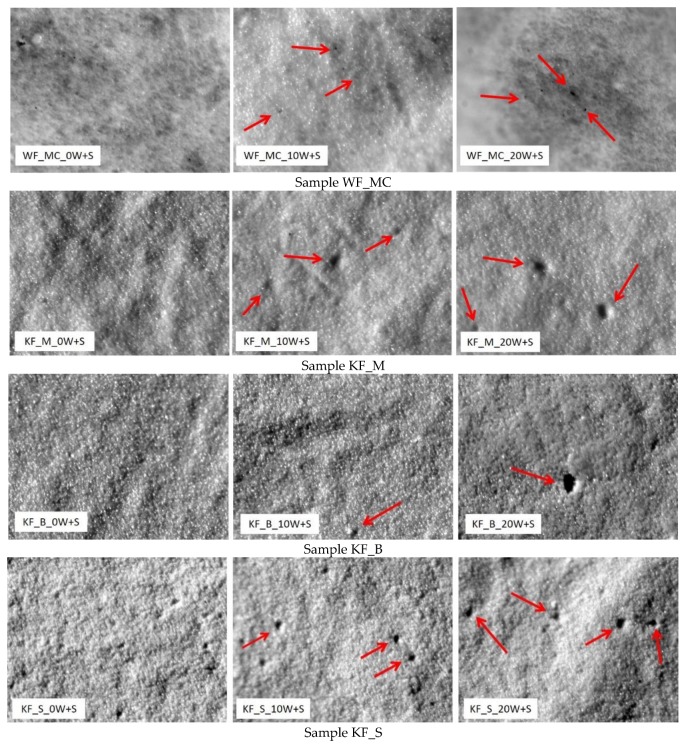
Microscopic analysis of the polyurethane coating, magnification 128×.

Microscopic analysis of the polyurethane coating before washing and sterilization procedures and after 10 and 20 washing and sterilization procedures is shown in Figure 5.

Microscopic analysis of the polyurethane coating surface shows damage to the PU coating that increases with increased frequency of washing and sterilization, Figure 5. The smallest surface damage is observed in the sample WF_MC in which the substrate is woven fabric and that has the largest mass per unit area. Samples KF_M show the largest damage, after only 10 W + S. The cause is assumed to be the smallest mass per unit area of 127 m^2^ and a sample thickness of 0.30 mm. Microscopic images of the KF_B sample show less damage to the PU layer but with the highest perforation. In the sample KF_S approximately the same damage to the surface layer is observed.

Table 4 shows the results of breaking force (N), tensile strength (N/mm) and breaking elongation (%) for samples WF_MC, KF_M, KF_B and KF_S without washing and sterilization (0 W + S) after 10 and 20 washing and sterilization cycles (10 W + S, 20 W + S).

**Figure 6:**
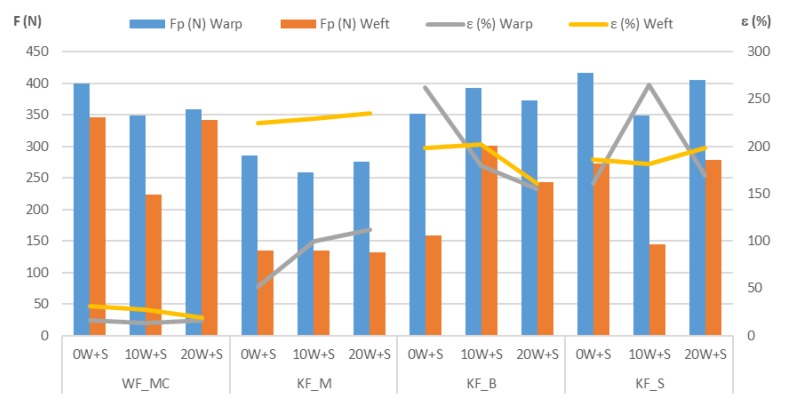
Results of breaking forces (F/N) and breaking elongation (ε/%).

**Figure 7:**
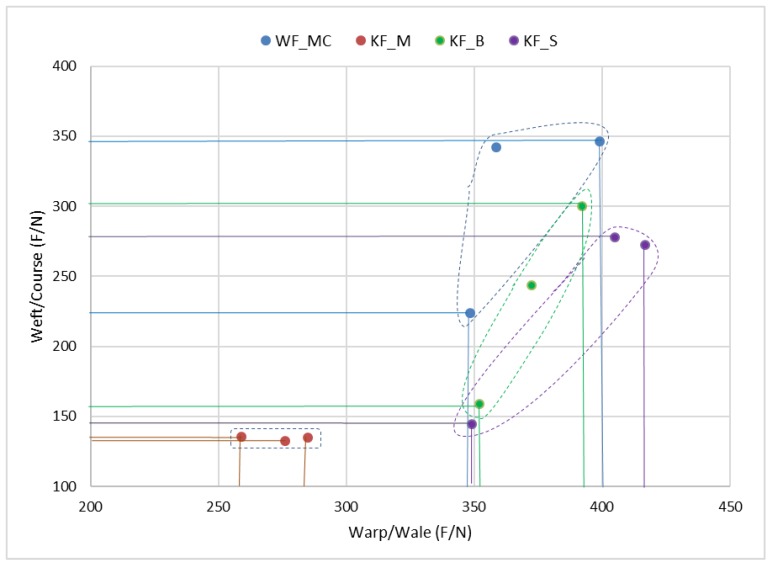
Relations of the breaking forces in the warp/wale and weft/course direction for each sample.

**Figure 8:**
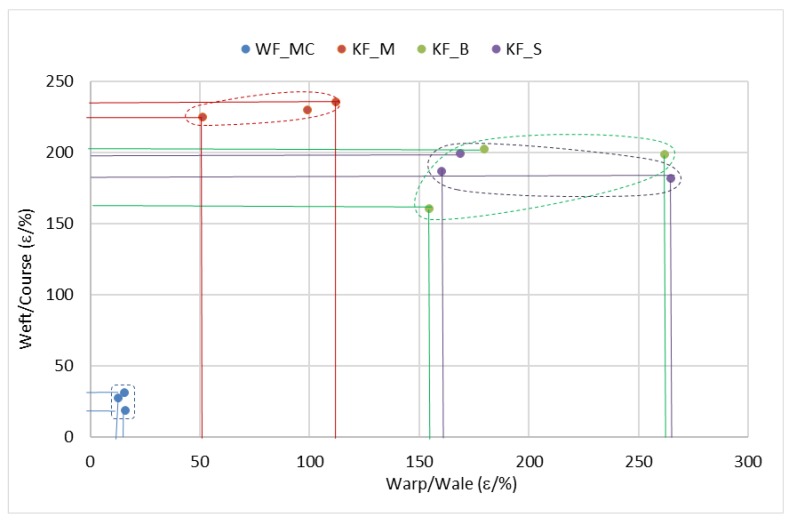
Relations of the breaking elongation in the warp/wale and weft/course direction for each sample.

Mechanical damage to medical textiles in the washing and sterilization processes was determined according to standard EN ISO 13934-1 1999 by determining breaking force F (N) and breaking elongation ε (%) (Strength Tester MesdanLab). The measuring length of the test specimens was 100 mm with the elongation speed at 100 mm/min. The influence of the washing and sterilization procedures on the breaking forces and breaking elongation of the samples are shown in Table 4 and the comparison of the samples in Figure 6, Figure 7 and Figure 8.

The relations between the breaking forces in the warp/wale direction and the weft/course direction for each sample are shown in Figure 7. According to the scattering values in the coordinate system, it can be seen that the sample KF_M deviates from the other samples. It is the sample having the lowest breaking forces in the wale and course direction. The other three samples have almost the same scattering area of breaking forces in the warp/wale direction, while in the weft/course direction the sample WF_MC with the woven fabric has slightly higher values than the other two samples.

Figure 8 shows the relation of the breaking elongations in the warp/wale direction and the weft/course direction and their scattering values. Sample WF_MC with the woven fabric in the substrate has very narrow scattering values and stands out with the lowest breaking elongation in the warp and weft direction. The other three samples with the knitted fabric in the substrate have significantly higher breaking elongation and greater scattering area, especially in the wale direction. However, sample KF_M stands out with the highest breaking elongations in the course direction compared to the other two samples with the knitted fabric in the substrate (KF_B and KF_S) which actually overlap in the areas of scattering values.

Statistical analysis of samples (ANOA) is presented in Table 5 and Table 6. Table 5 includes the parameters of the breaking forces in the warp and weft direction with the sample thickness and mass. According to the obtained F_stat_ the significance of results (F_stat_. > F_tab_. → 8.8419 > 4) for the alpha level selected (0.01) is evident.

Table 6 includes the parameters of breaking forces in the warp and weft direction with the sample thickness and mass. According to the obtained F_stat_ the significance of results (F_stat_. > F_tab_. → 5.42867 > 4) for the alpha level selected (0.01) is also evident.

Table 7 lists the number of microorganisms on the fabric face side and the number of penetrated microorganisms on the back side for all four samples. The number of microorganisms on the face side represents the number of microorganisms retained after rubbing with a stick of bacterial endospores of the apathogenic species of the genus *Bacilllus Geobacillus Stearothermophilus 10*^5^ and *Bacillus Atrophaeus 10*^6^ over the surface or the face side of the sample.

The results show that in none of the samples the microorganism permeability to the back side occurred, meaning that the samples were impermeable for microorganisms. One of the reasons for the impermeability of the microbial barrier is the polyurethane coating present in all samples. The results show a change in the PU coating after the washing and sterilization procedures because of a visible increase in the number of microorganisms retained on the polyurethane coating. It can be concluded that the changes or damage caused by the washing and sterilization procedures on the PU coating resulted in a rougher surface. Rougher surface has the ability to retain more microorganisms, as revealed by the results obtained, Table 7. In sample WF_MC the quantity of received microorganisms (218 CFU) on the surface increased by 2.7 times compared to the initial value (82 CFU). In sample KF_M the number of microorganisms retained on the surface increased by 3.7 times (93 CFU). The highest increase in the number of microorganisms retained on the surface by 3.9 times occurred in sample KF_B, while the number of received microorganisms on the surface of sample KF_S increased by 3.3 times (187 CFU) in comparison to the initial value (56 CFU). It can be concluded that the smallest increase in retained microorganisms on the PU coating occurred in sample of WF_MC whose substrate is woven fabric. Since this sample has woven fabric as the stronger substrate, remaining more stable during the washing and sterilization processes, only a slight change occurred in the polymer coating.

The durability or retention period of the microbial barrier of sterilized diagonally packed packages (single layer; EN ISO 11607-1 2009), was determined after storage. The testing results of the durability and effectiveness of the microbial barrier under controlled storage conditions (microclimatic conditions: temperature 15–30 °C and relative humidity 30–60%) over a period of 1, 2 and 3 months after a series of 10 and 20 washing and sterilization procedures show appropriate durability of the microbial barrier. No contamination of the package contents of any of the tested samples occurred. The impermeable microbial barrier is very important for medical textile materials used to package and store sterile instruments, surgical gowns and covers to separate the sterile area from the non-sterile one, etc.

### 3.1. FTIR–ATR Analysis of the Polyurethane Coating

**Figure 9:**
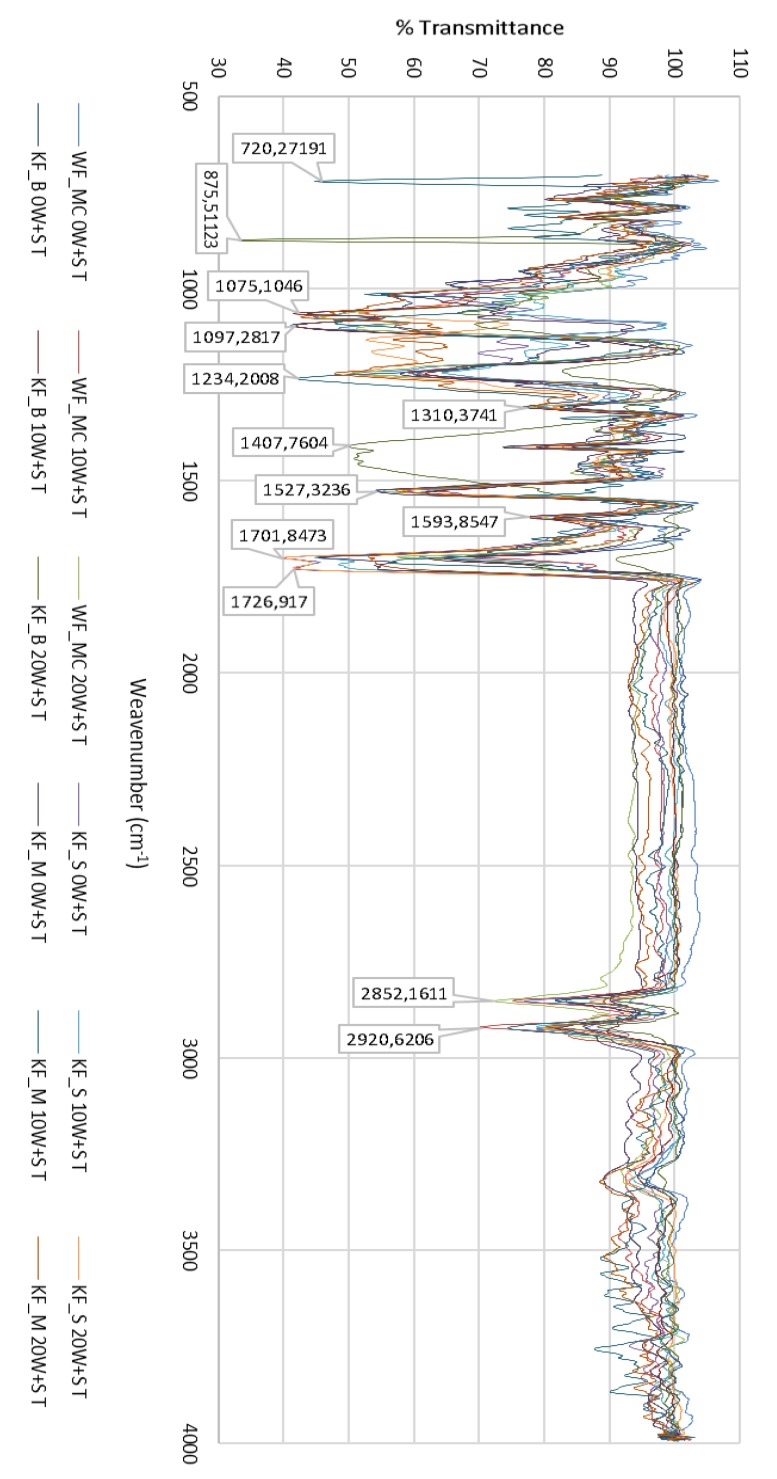
Entire FTIR spectrum of the PU coating of all samples: WF_MC, KF_S, KF_B, KF_M for 0 W + S, after 10 W + S and after 20 W + S.

FTIR analysis of unwashed and washed samples of PU-coated, CO (Cotton), PA (Polyamide) and PES (Polyester) revealed changes in the shape and size of individual peaks, splitting of peaks, changes in the ratio of the size of two adjacent peaks, etc. The greatest changes were found in the peaks corresponding to the urethane bond and the bond of ester oxygen with adjacent atoms. Changes are more noticeable with the number of washes or with the extension of aging time, Figure 9.

**Figure 10:**
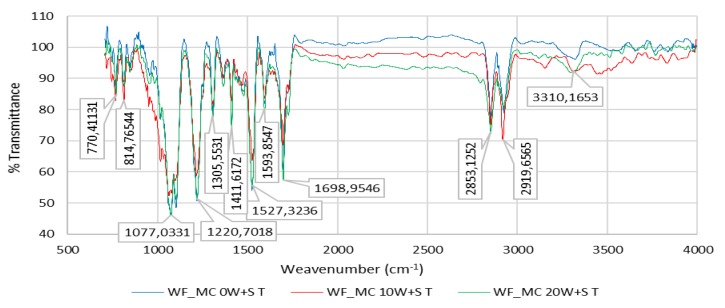
FTIR spectrum of the PU coating of sample WF_MC (40%/CO 60%) for 0 W + S, after 10 W + S and after 20 W + S.

Figure 10 shows the FTIR spectrum of the PU coating of sample WF_MC (PU 40%/CO 60%) for 0 W + S, after 10 W + S and after 20 W + S.

### 3.2. FTIR Spectrum of the PU Coating of Sample WF_MC (40%/CO 60%) for 0 W + S

The FTIR spectrum of the coating agent made from PU (100% PU) showed the characteristic peaks of PU, such as peaks of carbonyl groups *ν*(C=O) at 1717–1728 cm^−1^, amide III groups *δ*(O=C-N) at 1220 cm^−1^ and overlapped peaks of hydroxyl group *ν*(O-H) and amine group ν(N-H) at 3322 cm^−1^. The broad and strong peak ranging from 3200 to 3300 cm^−1^ and assigned to *ν*(OH) groups showed a change in shape and position on the spectrum after washing (mechanical) pretreatment and was ascribed to the intramolecular and intermolecular hydrogen bonds of cellulose. Schwanninger et al. reported that the O–H stretching vibration region became broader during mechanical washing because of the breakage of the hydrogen bonds of cellulose [43].

### 3.3. FTIR Spectrum of the PU Coating of Sample WF_MC (40%/CO 60%) after 10 W + S

FTIR-ATR of PU-treated cotton fabrics shows the principal spectral character of cotton fabric: the peaks round 3321, 2920 and 1070 cm^−1^ are assigned to the stretching of *ν*(OH), *ν*(CH) and *δ*(C–O–C) deformation vibration in the cellulosic structure, respectively. The FTIR spectrum of the cotton fabric exhibited a characteristic broad peak at 3321 cm^−1^ corresponding to *ν*(OH) stretching vibration, confirming the existence of hydrogen bonding of cellulose. Peaks at 2920 cm^−1^ and 1307 cm^−1^ belong to *ν*(CH) symmetric stretching and *δ*(CH) bending vibration bands, respectively. In addition, the absorption band round 1160 cm^−1^ (are not noticeable as they are masked by absorption of stronger functional groups of polyurethane), correspond to *β* (1→4) glycosidic linkages of *ν*(C–O–C) stretching in cellulose polymer of cotton fiber.

### 3.4. FTIR Spectrum of the PU Coating of Sample WF_MC (40%/CO 60%) after 20 W + S

Figure 5 shows the elongation of the cotton fabric after coating with PU, indicating an interaction between hydroxyl groups of cellulose and carbonyl groups (>C=O) of polyurethane. It can be suggested that the chemical interaction through H-bonding was responsible for the interfacial adhesion between PU and cotton fabrics after coating. Additionally, the change in absorbance at *ν*(OH) stretching vibration of the cotton fabric after coating with PU indicates a chemical interaction between carbonyl groups (>C=O) of PU and hydroxyl groups (−OH) of cellulose polymer [25]. This chemical interaction suggests a strong interfacial adhesion between PU and cotton fabrics after coating through H-bonding, i.e., any formation of an intermolecular hydrogen bond moves the bands relative to the base position and thus the band is batochromically shifted.

**Figure 11:**
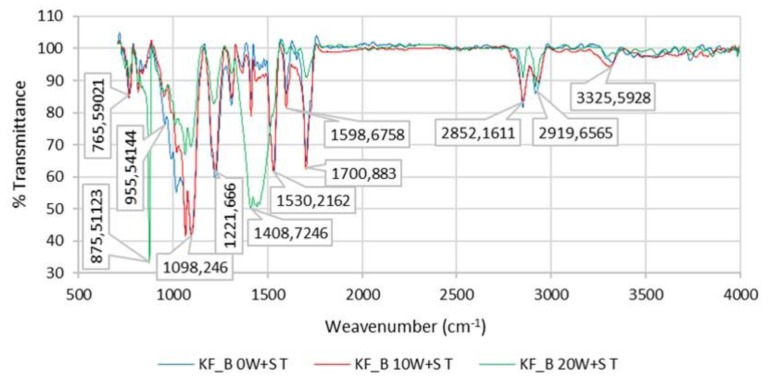
FTIR spectrum of the PU coating of sample KF_B (PU 47%/PA 53%) for 0 W + S, after 10 W + S and after 20 W + S.

Figure 11 shows FTIR spectrum of the PU coating of sample KF_B (PU 47%/PA 53%) for 0 W + S, after 10 W + S and after 20 W + S.

### 3.5. FTIR Spectrum of the PU Coating of Sample KF_B (PU 47%/PA 53%) for 0 W + S, after 10 W + S and after 20 W + S

The spectrum obtained using an ATR-FTIR spectrometer shows polyamide-specific bands at: 3302–3324 cm^−1^ for *ν*(N–H) valence vibration; 2918–2931 cm^−1^ for the asymmetric valence vibration *ν*_as_(CH_2_); 2850 cm^−1^ for *ν*_s_(CH_2_) valence vibration; at 1703 cm^−1^ for *ν*(C=O) valence vibration; 1651 cm^−1^ for an amide band I; 1531 cm^−1^ for *δ*(N–H) deformation vibration; 1456 cm^−1^ for *δ* (CH_2_) symmetric deformation vibration; 1215–1220 cm^−1^ for ν(C–O–C) valence vibration; and at 1096 cm^−1^ for *ν*(C–N) valence vibration (Figure 6). All amides are characterized by the absorption of bands known as amide band I, which occurs in the range 1630–1680 cm^−1^.

The amide II band usually occurs at about 1598–1600 cm^−1^ (in this case, the amide II band has a higher intensity than the amide I band due to the influence of the surrounding functional groups, i.e., interaction with them).

**Figure 12:**
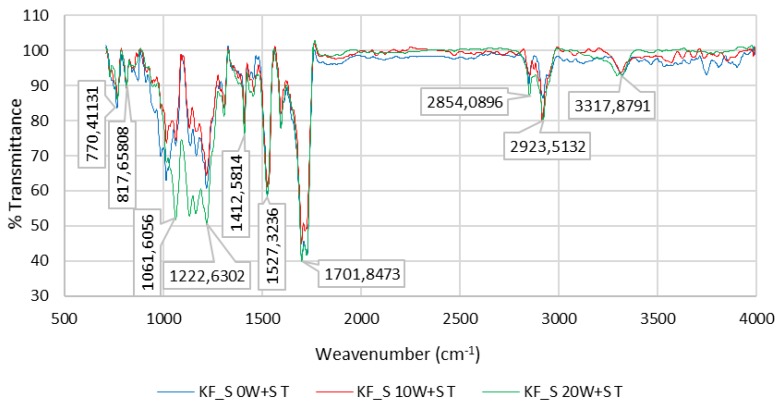
FTIR spectrum of the PU coating of sample KF_S (PU 50%/PES 50%) for 0 W + S, after 10 W + S and after 20 W + S.

**Figure 13:**
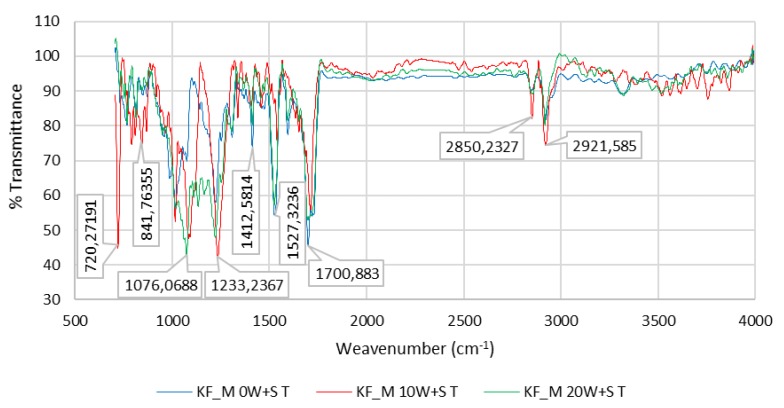
FTIR spectrum of the PU coating of sample KF_M (PU 58%/PES 42%) for 0 W + S, after 10 W + S and after 20 W + S.

Figure 12 and Figure 13 show the FTIR spectra of the PU coating of sample KF_S (50%/PA 50%) and the PU coating of sample KF_M (58%/PES 42%) for 0 W + S, after 10 W + S and after 20 W + S.

Characteristic poly (ethylene terephthalate) (PET) bands can be seen after ATR-FTIR spectroscopy. They occur at 2922 cm^−1^ where *ν*(C–H) valence vibration occurs on an aromatic ring; the peaks at 1717–1726 cm^−1^ can be assigned to *ν*(C=O) valence vibration; at 1411 cm^−1^ the valence vibration of the aromatic ring occurs; the peak at 1351 cm^−1^ belongs to *δ*(CH_2_) deformation vibration, at 1220 cm^−1^ *ν*(COO) valence vibration occurs; the peaks at 1064 cm^−1^ belong to *ν*(C–H) valence vibration; at 1017 cm^−1^ and 731 cm^−1^ *ν*(C–H) and *δ*(C=H) occur, Figure 12 and Figure 13.

## 4. Conclusions

The paper deals with the microbial barrier effectiveness of polyurethane coated woven and knitted fabrics with respect to washing rapidness and sterilization process. Bacterial spores of the *genus Bacillus* (*Geobacillus Stearothermophilus* and *Bacillus Atrophaeus*) were used. The reason is that those are the only form of microorganisms in the dry form and are also used in biological sterilization control.

This paper examines the microbial barrier effectiveness of textile packaging materials and determines the durability of the microbial barrier over a period of one, two and three months. The results showed that all samples had an effective microbial barrier during 20 washing and sterilization procedures. Additionally, the durability of the microbial barrier tested over three months showed that microorganisms do not penetrate the inside of the sterilized packages. It can be concluded that the samples have an extremely effective microbial barrier. The durability of the barrier was proven after 10 and 20 washing and sterilization procedures and after storage under strictly controlled conditions for a period of one, two and three months. The polyurethane coating in the range from 40 to 58% has a great influence on such an impermeable barrier. The test results of the microbial barrier permeability also showed a change on the PU coating surface. The increase in retained microorganisms on the PU coating surface with respect to the initial value ranged from 2.7 times to 3.9 times.

The washing and sterilization of samples often result in their permanent shrinkage and these changes cause a linear increase in mass and thickness. Breaking forces and breaking elongation are also changed by the washing and sterilization process, mostly to lower values, especially after 10 washing and sterilization procedures. Since the samples continue to shrink after 20 washes and sterilization, there is a “phenomenon” of increasing breaking forces and breaking elongation, meaning that the deformations created by washing and sterilization have less effect on breaking forces and breaking elongation compared to shrinkage.

The results of the FTIR analysis show a change in the polyurethane coating surface, which is more noticeable with a higher number of washes, or with the extension of aging time. Bacterial permeability was not observed on any sample after three months of storage and after 20 washing and sterilization procedures, suggesting that they were extremely effective.

## Figures and Tables

**Table 1:** Basic parameters of polyurethane coated fabrics.

Samples	Structure	Composition	Substrate/Raw Material Content	Layer/Coated	Mass Per Unit Area (g/m^2^)	Thickness (mm)
WF_MC	Plain	40% PU/60% CO	100% Cotton woven fabric	100% PU	258.70	0.44
KF_M	Plain	58% PU/42% PES	100% PES knitted fabric	100% PU	127.26	0.30
KF_B	Rib 1x1	47% PU/53% PA	100% PA knitted fabric	100% PU	194.15	0.52
KF_S	Rib 1x1	50% PU/50% PES	100% PES knitted fabric	100% PU	200.56	0.47

WF—woven fabric; KF—knitted fabric; CO—Cotton; PES—Polyester; PA—Polyamide.

**Table 2:** Washing parameters.

Washing Solution	Disinfecting Agent	Temperature, °C	Bath Ratio
0.7 g/kg Ce 2.5 g/kg Ca	4 g/kg Cc	60	1:5

* Commercial names of all products are not given due to the secrecy of the laundry washing participant identification and impartiality of the research. Ca-Polycarboxylate (<5%), sodium hydroxide (10–20%). Cc-etoxylated fat alcohol < C15 and < 5EO (25–30%), solvent, 2-propanol, methanol (0.1–0.25%), amphoteric surfactants (1–2%), additives (0.1–0.25%). Ce-formic acid (50–100%).

**Table 3:** Results of measuring construction parameters.

Samples		Mass Per Area (g/m^2^)			Thickness (mm)			* Dimensional Change (%)	
		0 W + S	10 W + S	20 W + S	0 W + S	10 W + S	20 W + S	10 W + S	20 W + S
WF_MC	Mean	258.70	258.35	250.09	0.44	0.46	0.44	625 cm^2^ 0%	623.13 cm^2^ −0.3%
	SD	1.07	4.29	1.82	0	0	0		
	CV (%)	0.41	1.66	0.73	1.15	2.9	2.0		
KF_M	Mean	127.26	136.05	136.01	0.30	0.34	0.34	575 cm^2^ −8%	575 cm^2^ −8%
	SD	1.72	0.23	3.15	0	0	0		
	CV (%)	1.35	0.17	2.32	1.0	2.0	2.9		
KF_B	Mean	194.15	203.33	209.00	0.52	0.55	0.58	600 cm^2^ −4%	587.50 cm^2^ −6%
	SD	1.32	3.97	0.01	0	0	0		
	CV (%)	0.68	1.95	0	0.9	1.9	1.4		
KF_S	Mean	200.56	215.96	216.13	0.47	0.50	0.51	568.75 cm^2^ −9%	568.75 cm^2^ −9%
	SD	2.6	0.42	2.73	0	0	0		
	CV (%)	1.3	0.19	1.26	3.2	1.8	2.3		

SD—standard deviation; CV—coefficient variation (%); W + S—washing and sterilization; WF—woven fabric; KF—knitted fabric; * Original Dimension (cm) 25 × 25 cm = 625 cm^2^.

**Table 4:** Results of breaking force (F/N), tensile strength (N/mm) and breaking elongation (ε/%).

Samples		Warp						Weft					
		F (cN)		T (N/mm)		ε (%)		F (cN)		T (N/mm)		ε (%)	
		Mean	CV (%)	Mean	CV (%)	Mean	CV (%)	Mean	CV (%)	Mean	CV (%)	Mean	CV (%)
WF_MC	0 W + S	399.01	5.90	18.14	3.81	15.80	5.73	346.19	7.75	15.74	6.65	30.84	3.53
	10 W + S	348.33	7.25	15.14	6.02	12.99	10.33	223.70	25.86	9.73	10.23	27.48	4.15
	20 W + S	358.75	12.23	16.31	10.33	16.16	9.17	342.18	10.88	15.55	8.99	18.45	1.80
KF_M	0 W + S	285.02	9.31	19.00	7.52	51.54	5.19	134.73	8.94	8.98	7.66	224.43	3.69
	10 W + S	258.95	5.78	15.23	4.54	99.27	2.89	135.36	2.84	7.96	2.05	229.65	4.40
	20 W + S	276.12	4.27	16.24	3.27	112.11	2.57	132.50	2.90	7.79	3.11	235.20	2.98
KF_B	0 W + S	352.21	2.27	13.55	1.99	158.98	2.68	262.13	2.34	10.08	2.98	198,08	1.82
	10 W + S	392.30	5.13	14.27	4.02	179.83	2.84	300.32	13.26	10.92	9.87	202.10	4.50
	20 W + S	372.5	9.11	12.84	7.56	154.69	4.37	243.54	10.49	8.40	8.73	160.50	4.70
KF_S	0 W + S	416.74	4.24	17.73	3.28	160.41	1.81	272.60	5.56	11.60	6.01	186.49	0.88
	10 W + S	348.96	3.98	13.96	2.07	144.48	1.94	264.81	6.74	10.59	6.51	181.71	5.52
	20 W + S	405.02	4.30	15.88	2.99	169.04	2.10	278.10	1.59	10.91	2.65	198.60	2.83

WF—woven fabric; KF—knitted fabric; CV—coefficient variation (%); W + S—washing and sterilization; T—tensile strength.

**Table 5:** Results of the ANOVA analysis for breaking force.

**Data Summary**								
**Groups**	**N**		**Mean**		**Std. Dev.**		**Std. Error**	
Group 1	24		22.7083		23.9809		4.8951	
Group 2	24		101.2917		106.0092		21.639	
Group 3	24		192.8333		167.4875		34.1882	
Group 4	24		136.5		123.2862		25.1657	
**ANOVA Summary**								
**Source**		**Degrees of Freedom** **DF**		**Sum of Squares** **SS**		**Mean Square** **MS**	**F-Stat**	***p*-Value**
Between Groups		3		365156.0819		121718.694	8.8419	0.00003
Within Groups		92		1266485.4278		13766.146		
**Total:**		95		1631641.5096				

The *f*-ratio value is 8.84189. The *p*-value is 0.000033. The result is significant at *p* < 0.01.

**Table 6:** Results of the ANOVA analysis for elongation at break.

**Data Summary**								
**Groups**	**N**		**Mean**		**Std. Dev.**		**Std. Error**	
Group 1	24		22.7083		23.9809		4.8951	
Group 2	24		101.2917		106.0092		21.639	
Group 3	24		76.125		59.436		12.1323	
Group 4	24		96.4583		86.9017		17.7387	
**ANOVA Summary**								
**Source**		**Degrees of Freedom** **DF**		**Sum of Squares** **SS**		**Mean Square** **MS**	**F-Stat**	***p*-Value**
Between Groups		3		93227.5484		31075.8495	5.42867	0.00175
Within Groups		92		526644.285		5724.3944		
**Total:**		95		619871.8334				

The *f*-ratio value is 5.42866. The *p*-value is 0.001754. The result is significant at *p* < 0.01.

**Table 7:** Results of microbial barrier permeability: the average number of bacterial colonies (CFU) on the back and face side.

Samples	Number of Isolate	The Average Number of Bacterial Colonies on the Face Side (CFU)			The Average Number of Bacterial Colonies on the Back Side (CFU)		
		0 W + S	10 W + S	20 W + S	0 W + S	10 W + S	20 W + S
WF_MC	6	82	162	218	0	0	0
KF_M	6	25	92	93	0	0	0
KF_B	6	44	173	171	0	0	0
KF_S	6	56	185	187	0	0	0

W + S—washing and sterilization; WF—woven fabric; KF—knitted fabric.
